# Serum Testosterone Concentrations and Urinary Bisphenol A, Benzophenone-3, Triclosan, and Paraben Levels in Male and Female Children and Adolescents: NHANES 2011–2012

**DOI:** 10.1289/EHP150

**Published:** 2016-07-06

**Authors:** Franco Scinicariello, Melanie C. Buser

**Affiliations:** Division of Toxicology and Human Health Sciences, Agency for Toxic Substances and Disease Registry (ATSDR), Atlanta, Georgia, USA

## Abstract

**Background:**

Exposure to environmental phenols (e.g., bisphenol A, benzophenone-3, and triclosan) and parabens is widespread in the population. Many of these chemicals have been shown to have anti-androgenic effects both in vitro and in vivo.

**Objective:**

We examined the association of bisphenol A (BPA), benzophenone-3 (BP-3), triclosan (TCS), and parabens with serum total testosterone (TT) levels in child and adolescent participants (ages 6–19 years) in the National Health and Nutrition Examination Survey (NHANES) 2011–2012.

**Methods:**

We performed multivariable linear regression to estimate associations between natural log–transformed serum TT and quartiles of urinary BPA, BP-3, TCS, and parabens in male and female children (ages 6–11 years) and adolescents (ages 12–19 years).

**Results:**

BP-3 and BPA were associated with significantly lower TT in male adolescents, and BPA was associated with significantly higher TT in female adolescents. TT was not consistently associated with TCS or total parabens in children or adolescents of either sex.

**Conclusions:**

To our knowledge, this is the first study to report an association of both BP-3 and BPA with serum TT in adolescents. Associations between BPA and TT differed according to sex in adolescents, with inverse associations in boys and positive associations in girls. BP-3 was associated with significantly lower TT in adolescent boys only. However, because of the limitations inherent to the cross-sectional study design, further studies are needed to confirm and elucidate on our findings.

**Citation:**

Scinicariello F, Buser MC. 2016. Serum testosterone concentrations and urinary bisphenol A, benzophenone-3, triclosan, and paraben levels in male and female children and adolescents: NHANES 2011–2012. Environ Health Perspect 124:1898–1904; http://dx.doi.org/10.1289/EHP150

## Introduction

Endocrine-disrupting compounds (EDCs) are synthetic and naturally occurring chemicals that may interfere with endogenous endocrine action; particularly, *in utero* exposure or exposure during puberty may produce adverse developmental, reproductive, neurological, and immune effects in both humans and wildlife. Exposure to EDCs has been associated with reproductive problems, obesity, diabetes, cancers, and behavioral and learning disorders (reviewed in WHO/UNEP 2013). Several substances are thought to cause endocrine disruption; most EDCs are classified as xenoestrogens whereas others inhibit androgen production and function (Meeker 2012). One class of EDCs is the environmental phenols, which includes bisphenol A (BPA), triclosan (TCS), and benzophenone-3 (BP-3). People are exposed to environmental phenols through industrial pollution, food consumption, and the use of personal care and consumer products. Parabens are another class of EDCs that are prevalent in the environment. These substances are used in cosmetics, pharmaceuticals, as antimicrobial preservatives against mold, and in food and beverage packaging (Calafat et al. 2010).

BPA is considered to have both estrogenic and anti-androgenic effects. In a recent review of the BPA literature, Peretz et al. (2014) concluded that BPA is a reproductive toxicant based on findings from experimental and epidemiological studies, with strong evidence that BPA is an ovarian toxicant in human females and animal models, and limited evidence of effects on other reproductive outcomes in men, women, and animals. In rat models, prenatal or perinatal BPA exposure has been shown to have adverse effects on the male reproductive system including decreased epididymal weight and reduced testicular sperm counts (Salian et al. 2009a, 2009b). Additionally, decreased testosterone (T) levels have been observed in rodents exposed to BPA during the prepubertal (Takao et al. 1999) and pubertal period (Herath et al. 2004). The available body of literature suggests that BPA is probably a testicular toxicant in animals, but the data in humans are more ambiguous (Peretz et al. 2014).

TCS has demonstrated anti-androgenic action in *in vitro* studies. Competitive binding assays with a recombinant androgen receptor (AR) have shown that TCS binds to the receptor with relatively high avidity (Gee et al. 2008). The TCS–AR interaction was also confirmed in the T47D human breast cancer cell line (Ahn et al. 2008).

The evidence for reproductive effects following exposure to BP-3 has been less extensively investigated. We identified a single panel study of 15 young men (age 23–29 years) and 17 postmenopausal women (age 54–86 years) that compared average levels of reproductive hormones after topical application of cream containing BP-3 (10% wt/wt) to levels after application of a control cream. Although some differences were observed, the authors concluded these were probably chance findings (Janjua et al. 2004). Evidence for reproductive effects comes largely from *in vitro* studies that have demonstrated both estrogenic and anti-androgenic effects of BP-3 (reviewed by Krause et al. 2012). Moreover, some experimental animal studies have looked at BP-3, but these focus mostly on adult animals with few studies looking at early development (reviewed by Krause et al. 2012).

*In vivo* studies of the effect of parabens on testosterone levels have produced mixed results. Rats and mice exposed *in utero* to parabens showed a significant reduction of testosterone levels and mature sperm counts (Kang et al. 2002; Oishi 2002a, 2002b), whereas other studies found no significant changes (Hoberman et al. 2008; Taxvig et al. 2008).

Data from the National Health and Nutrition Examination Survey (NHANES), collected by the Centers for Disease Control and Prevention (CDC), have shown widespread exposure to BPA, BP-3, TCS, and parabens among the U.S. general population, including children and adolescents. The NHANES 2011–2012 cycle includes serum total testosterone (TT) measurements in participants ≥ 6 years of age. Because of existing concerns about the anti-androgenic activity of environmental phenols and parabens, we evaluated the relationship of TT with urinary levels of BPA, BP-3, TCS, and total parabens (ΣPAR) among male and female children (ages 6–11 years) and adolescents (ages 12–19 years) in a representative U.S. population sample.

## Method

### Study Population

NHANES is a cross-sectional, nationally representative survey of the noninstitutionalized civilian population of the United States conducted annually by the National Center for Health Statistics (NCHS), CDC (Johnson et al. 2013). For our study, we used the publicly available files for NHANES cycles 2011–2012. The survey employs a multistage stratified probability sample based on selected counties, blocks, households, and persons within households.

NCHS-trained professionals conducted interviews in participants’ homes; extensive physical examinations, including blood and urine collection, were conducted at mobile examination centers. All procedures were approved by the NCHS Research Ethics Review Board (Continuation of Protocol #2011-17; http://www.cdc.gov/nchs/nhanes/irba98.htm), and all participants provided written informed consent. In the 2011–2012 data set, urinary concentrations of environmental phenols and parabens were measured in a randomly selected one-third subsample of persons 6 years of age and older by the CDC’s National Center for Environmental Health (NCEH), Division of Laboratory Sciences (DLS), which coordinates the National Biomonitoring Program (NBP) to assess nutritional status and exposure of the U.S. population to environmental chemicals and toxic substances. For our analysis, we included only those participants who had measurements for urinary phenols and parabens and information regarding the covariates included in the model (*n* = 588).

### Serum Total Testosterone

Serum samples were shipped to the CDC/NCEH/DLS, where the serum TT was analyzed by isotope dilution liquid chromatography tandem mass spectrometry. Information regarding reliability, validation, and quality control for serum TT are described in the NHANES laboratory method (http://www.cdc.gov/nchs/data/nhanes/nhanes_11_12/TST_G_met.pdf). Serum TT was natural log–transformed for analyses because the distribution of this variable was skewed left.

### Urinary Biomarkers

Spot urine samples were collected from study participants and stored at –20°C; they were then analyzed by NCEH/DLS. BPA, BP-3, TCS, methyl paraben (MPB), and propyl paraben (PPB) were measured by solid phase extraction coupled on-line to high performance liquid chromatography and tandem mass spectrometry. Details of detection and measurement of the urinary compounds are described in the NHANES laboratory methods (http://www.cdc.gov/nchs/nhanes/nhanes2011-2012/lab_methods_11_12.htm). The reported results for all assays meet the NCEH/DLS quality control and quality assurance performance criteria for accuracy and precision (NCHS 2013). The limits of detection (LOD) were 0.4 ng/mL for BPA, 0.4 ng/mL for BP-3, 2.3 ng/mL for TCS, 1.0 ng/mL for MPB, and 0.2 ng/mL for PPB. Urinary concentrations of the compounds below the level of detection were assigned the limit of detection divided by the square root of 2, as recommended by NHANES (NCHS 2013). To account for variation in dilution in spot urinary samples, we adjusted for urinary creatinine by including it as a model covariate (Barr et al. 2005; Ikeda et al. 2003). The parabens (ΣPAR) were considered together as a sum of total parabens (MPB and PPB), calculated by adding the concentration of each individual metabolite (Buttke et al. 2012). There were no significant correlations between the urinary compounds (data not shown), except between urinary ΣPAR and TCS or BPA as indicated by the Pearson correlation coefficients (*r* = 0.18, *p* < 0.0001; and *r* = 0.08 *p* < 0.04, respectively).

### Covariates

All models were adjusted for the following *a priori* covariates: age, race/ethnicity, urinary creatinine, poverty income ratio (PIR), obesity, season of collection, time of venipuncture, total serum cholesterol, serum cotinine as a biomarker of exposure to environmental tobacco smoke, and physical activity (only for male and female adolescents). Race/ethnicity was categorized as non-Hispanic white, non-Hispanic black, Hispanic (Mexican American and other Hispanic), and other. PIR is a measure of socioeconomic status and represents the calculated ratio of household income to the poverty threshold after accounting for inflation and family size; it was entered as a continuous variable. Time of venipuncture is found in the NHANES Fasting Questionnaire File and was classified as morning, afternoon, or evening sessions. Season of collection was obtained from the NHANES demographic data pertaining to the 6 month time period when the examination occurred; this was either 1 November–30 April or 1 May–31 October. We included this covariate because of the differences in use of these products (i.e., use of sunscreen with BP-3 and parabens increases during the summer). Moreover, levels of TT in men have a wide range of variation due to diurnal, weekly, and seasonal variations (Brambilla et al. 2009; Cunningham and Toma 2011). Children and adolescents were classified as underweight, normal weight, overweight, or obese according to age and sex, as defined by NHANES (Body Measures File; http://wwwn.cdc.gov/Nchs/Nhanes/2011-2012/BMX_G.htm). For our analyses, we combined underweight and normal weight as one category. Serum cotinine and urinary creatinine were natural log–transformed. Information on recreational physical activity was available for adolescents only; participants were asked whether they engaged in regular moderate and/or vigorous recreational activities (categorized as yes or no).

In addition to models adjusted for the covariates indicated above, we ran a second model for each exposure that was adjusted for urinary concentrations of the other three exposures, which were modeled as natural log–transformed continuous variables.

### Statistical Methods

All analyses were performed using the weight from the urinary phenols and parabens subsample, as recommended by NCHS, to account for the complex sampling design and nonresponse of NHANES; all analyses were performed according to NHANES guidelines (Johnson et al. 2013). SAS 9.3 (SAS Institute Inc., Cary, NC) was used for all statistical analyses, and SAS-Callable SUDAAN 10 (Research Triangle Institute, Research Triangle Park, NC) was used to account for the NHANES complex sample design. *p*-Values were presented at the significance level < 0.05.

Analyses were conducted for male children (6–11 years), male adolescents (12–19 years), female children (6–11 years), and female adolescents (12–19 years). We used multivariable linear regression to calculate adjusted β coefficients for the associations between natural log–transformed TT and urinary phenol and paraben levels among the participants. Urinary BPA, BP-3, TCS, and ΣPAR were categorized according to quartiles defined for each population subgroup.

Because our dependent variable, TT, was log-transformed, the results were retransformed by exponentiation of the β coefficients and presented as percent differences estimated by comparing each of the upper three quartiles to the lowest quartile. Statistical tests for linear trends were conducted by modeling quartiles as an ordinal variable using integer values.

## Results

Table 1 presents the characteristics of the study population by age and sex. The geometric mean (GM) serum TT levels for male children, male adolescents, female children, and female adolescents were 3.75 ng/dL, 276.07 ng/dL, 5.80 ng/dL, and 22.78 ng/dL, respectively (Table 1). There was a statistically significant difference in mean urinary TCS and ΣPAR levels between female children and female adolescents (*p* < 0.05) (data not shown). There were no other statistically significant differences in urinary compound levels between age- and sex-specific groups (e.g., differences in mean urinary levels between male children and adolescents, differences in mean urinary levels between male and female adolescents).

**Table 1:** Weighted characteristics for children and adolescent (6–19 years) participants in NHANES 2011–2012.

Characteristic	Male children	Male adolescents	Female children	Female adolescents
*n*
Urinary bisphenol A (ng/mL) [GM (SE)]	1.74 (0.26)	1.94 (0.29)	1.40 (0.17)	1.49 (0.21)
Urinary benzophenone-3 (ng/mL) [GM (SE)]	15.57 (4.61)	20.03 (5.69)	18.31 (3.96)	35.59 (14.40)
Urinary triclosan (ng/mL) [GM (SE)]	8.79 (1.80)	8.55 (1.67)	6.30 (0.72)	12.27 (3.05)
Urinary total parabens (ng/mL) [GM (SE)]	15.07 (4.19)	18.51 (4.15)	29.51 (6.58)	63.75 (17.23)
Age (years) [GM (SE)]	8.39 (0.20)	15.50 (0.19)	8.76 (0.15)	15.20 (0.17)
Serum total testosterone (ng/dL) [GM (SE)]	3.75 (0.60)	276.07 (27.71)	5.80 (0.54)	22.78 (0.73)
Serum cotinine (ng/mL) [GM (SE)]	0.06 (0.01)	0.12 (0.02)	0.05 (0.01)	0.14 (0.05)
Urinary creatinine (mg/dL) [GM (SE)]	71.18 (6.16)	112.65 (10.23)	68.09 (5.42)	98.87 (11.42)
Total cholesterol (mg/dL) [GM (SE)]	156.25 (2.27)	148.93 (2.61)	156.42 (3.33)	159.16 (5.82)
Ratio family income to poverty [GM (SE)]	1.65 (0.09)	1.73 (0.25)	1.76 (0.18)	1.73 (0.22)
Obesity^*a*^
Normal/underweight [% (SE)]	93.83 (1.93)	59.12 (3.11)	86.16 (3.16)	64.21 (8.17)
Overweight [% (SE)]	5.14 (2.31)	23.40 (3.64)	9.07 (3.85)	16.25 (4.16)
Obese [% (SE)]	1.03 (0.77)	17.49 (4.58)	4.78 (1.85)	19.54 (6.14)
Race/ethnicity
White (non-Hispanic) [% (SE)]	54.45 (5.59)	55.06 (4.75)	55.13 (7.84)	55.46 (6.60)
Non-Hispanic black [% (SE)]	15.40 (1.97)	15.73 (4.07)	12.21 (3.17)	15.44 (5.05)
Hispanic [% (SE)]	21.78 (5.12)	22.21 (3.98)	25.10 (5.25)	21.69 (4.07)
Other [% (SE)]	7.37 (2.49)	7.00 (2.45)	7.57 (2.12)	7.41 (1.44)
Session time of venipuncture
Morning	42.20 (5.42)	51.94 (6.77)	53.74 (4.01)	42.36 (4.89)
Afternoon	31.88 (3.96)	29.45 (4.98)	30.16 (4.51)	36.83 (4.64)
Evening	25.92 (4.75)	18.61 (5.37)	16.10 (3.06)	20.81 (5.11)
Six month time period when the examination was performed
1 November through 30 April [% (SE)]	52.60 (7.30)	47.64 (9.29)	48.08 (6.93)	40.15 (7.91)
1 May through 31 October [% (SE)]	47.40 (7.30)	52.36 (9.29)	51.92 (6.93)	59.85 (7.91)
Physical activity^*b*^
Yes [% (SE)]		90.23 (3.05)		71.43 (4.21)
No [% (SE)]		9.77 (3.05)		28.57 (4.21)
^***a***^Children and adolescents were classified as underweight, normal weight, overweight, or obese according to age and sex, as defined by NHANES (http://wwwn.cdc.gov/Nchs/Nhanes/2011-2012/BMX_G.htm). ^***b***^Information on recreational physical activity was available for adolescents only; participants were asked whether they engaged in regular moderate and/or vigorous recreational activities (categorized as yes or no).				

***Benzophenone-3*** (Table 2). Male adolescents in the 3rd and 4th quartiles of BP-3 had significantly lower TT [–38.74%; 95% confidence interval (CI): –58.52%, –10.42%; and –36.87%; 95% CI: –59.34%, –1.98%, respectively, based on model 1] than males in the lowest quartile. Although the association was strongest for the 3rd quartile, the overall trend was significant (*p*-trend = 0.01). This pattern of associations persisted following adjustment for BPA, TCS, and ΣPAR (model 2). In female adolescents, TT was significantly higher for girls in the second versus first quartile of BP-3 exposure, but positive associations were closer to the null and nonsignificant for the 3rd and 4th quartiles of exposure (*p*-trend 0.14). Associations were similar after adjustment for BPA, TCS, and ΣPAR, but no longer significant for quartile 2. There were no significant associations between TT and BP-3 in male or female children, and no evidence of consistent trends with increasing quartiles of exposure.

**Table 2:** Percent differences (95% CI) in serum total testosterone by quartiles of BP-3 exposure, National Health and Nutrition Examination Survey, 2011–2012.

BP-3 exposure	Model 1^*a*^	Model 2^*b*^
Male children
Q1: ≤ 3.5 ng/mL	Reference	Reference
Q2: 3.6–8.8 ng/mL	–1.98 (–51.81, 97.39)	7.25 (–46.74, 115.98)
Q3: 8.9–71.1 ng/mL	27.12 (–20.55, 105.44)	31.00 (–25.17, 129.33)
Q4: > 71.7 ng/mL	–1.98 (–44.57, 73.33)	4.08 (–40.55, 82.21)
*p*-Trend^*c*^	0.53	0.70
Male adolescents
Q1: ≤ 4.0 ng/mL	Reference	Reference
Q2: 4.1–15.9 ng/mL	–7.69 (–43.45, 50.68)	1.01 (–34.30, 55.27)
Q3: 16.0–48.7 ng/mL	–38.74 (–58.52, –10.42)	–32.97 (–54.16, –1.98)
Q4: > 48.7 ng/mL	–36.87 (–59.34, –1.98)	–32.97 (–54.16, –0.50)
*p*-Trend^*c*^	0.01	0.04
Female children
Q1: ≤ 6.6 ng/mL	Reference	Reference
Q2: 6.7–13.9 ng/mL	10.52 (–19.75, 50.68)	7.25 (–31.61, 68.20)
Q3: 14.0–58.5 ng/mL	5.13 (–20.55, 39.10)	–0.50 (–50.84, 101.38)
Q4: > 58.6 ng/mL	20.92 (–28.82, 105.44)	10.52 (–68.65, 293.54)
*p*-Trend^*c*^	0.87	0.91
Female adolescents
Q1: ≤ 8.6 ng/mL	Reference	Reference
Q2: 8.7–23.7 ng/mL	20.92 (3.05, 43.33)	17.35 (–1.00, 39.10)
Q3: 23.8–164.0 ng/mL	10.52 (–18.13, 49.18)	12.75 (–16.47, 50.68)
Q4: > 164.0 ng/mL	11.63 (–23.66, 64.87)	16.18 (–21.34, 71.60)
*p*-Trend^*c*^	0.14	0.27
^***a***^Adjusted for age, race/ethnicity, serum cotinine, urinary creatinine, total cholesterol, income, obesity, season of collection, time of venipuncture, and physical activity (for adolescents only). ^***b***^Adjusted for model 1 covariates plus natural log–transformed BPA, TCS, and ∑PAR. ^***c***^*p*-Value for exposure quartiles modeled as an ordinal variable using integer values.		

***Bisphenol A*** (Table 3). Mean TT was lower for all quartiles of exposure above the reference level in male adolescents (*p*-trend = 0.01), with significant associations for the 2nd and 4th quartiles of BPA (–49.34%; 95% CI: –70.18%, –13.06%; and –53.70%; 95% CI: –70.77%, –26.66% respectively, model 1). By contrast, TT was positively associated with BPA exposure in female adolescents, with a monotonic increase in mean TT with increasing quartiles of exposure (*p*-trend = 0.01) and significantly higher TT for the 4th quartile (53.73%; 95% CI: 9.42%, 113.83%) compared with the lowest quartile. BPA did not appear to be associated with TT in male or female children. Estimated associations and overall patterns were similar for all population subgroups after adjustment for BP-3, TCS, and ΣPAR.

**Table 3:** Percent differences (95% CI) in serum total testosterone by quartiles of BPA exposure, National Health and Nutrition Examination Survey, 2011–2012.

BPA exposure	Model 1^*a*^	Model 2^*b*^
Male children
Q1: ≤ 0.8 ng/mL	Reference	Reference
Q2: 0.9–1.5 ng/mL	16.18 (–44.01, 141.09)	9.42 (–46.74, 124.79)
Q3: 1.6–3.3 ng/mL	–14.79 (–62.84, 95.42)	–18.94 (–66.38, 93.48)
Q4: > 3.3 ng/mL	–10.42 (–41.14, 37.71)	–22.12 (–55.07, 34.99)
*p*-Trend^*c*^	0.27	0.19
Male adolescents
Q1: ≤ 0.9 ng/mL	Reference	Reference
Q2: 1.0–2.0 ng/mL	–49.34 (–70.18, –13.06)	–50.84 (–71.92, –13.93)
Q3: 2.1–3.7 ng/mL	–36.87 (–62.47, 6.18)	–38.12 (–63.21, 4.08)
Q4: > 3.7 ng/mL	–53.70 (–70.77, –26.66)	–52.29 (–71.35, –20.55)
*p*-Trend^*c*^	0.01	0.02
Female children
Q1: ≤ 0.7 ng/mL	Reference	Reference
Q2: 0.8–1.4 ng/mL	8.33 (–28.82, 64.87)	18.53 (–36.24, 118.15)
Q3: 1.5–2.8 ng/mL	8.33 (–30.93, 69.89)	19.72 (–42.88, 153.45)
Q4: > 2.8 ng/ mL	20.92 (–35.60, 127.05)	40.49 (–56.83, 352.67)
*p*-Trend^*c*^	0.86	0.94
Female adolescents
Q1: ≤ 0.7 ng/mL	Reference	Reference
Q2: 0.8–1.5 ng/mL	24.61 (–7.69, 69.89)	27.21 (–4.88, 71.60)
Q3: 1.6–3.2 ng/mL	39.10 (–4.88, 103.40)	36.34 (–6.76, 101.38)
Q4: > 3.2 ng/mL	53.73 (9.42, 113.83)	53.73 (12.75, 109.59)
*p*-Trend^*c*^	0.01	< 0.01
^***a***^Adjusted for age, race/ethnicity, serum cotinine, urinary creatinine, total cholesterol, income, obesity, season of collection, time of venipuncture, and physical activity (for adolescents only). ^***b***^Adjusted for model 1 covariates plus natural log–transformed BP-3, TCS, and ∑PAR. ^***c***^*p*-Value for exposure quartiles modeled as an ordinal variable using integer values.		

***Triclosan*** (Table 4). Mean TT was lower for all quartiles of TCS compared with the lowest quartile in all population subgroups, and trend *p*-values were significant for male children and adolescents (*p*-trend = 0.02 and 0.04, respectively). However, model 1 quartile-specific decreases were significant only for the 3rd quartile in male adolescents and in female children, and mean values were closest to the null for the highest quartile in male children and adolescents and in female children, whereas all associations were close to the null for female adolescents. Associations were generally consistent with model 1 estimates after adjustment for BP-3, BPA, and ΣPAR, though the negative association with quartile 3 was significant only in female children, and the *p*-trend was significant only for male children.

**Table 4:** Percent differences (95% CI) in serum total testosterone by quartiles of TCS exposure, National Health and Nutrition Examination Survey, 2011–2012.

TCS exposure	Model 1^*a*^	Model 2^*b*^
Male children
Q1: ≤ 2.5 ng/mL	Reference	Reference
Q2: 2.6–6.1 ng/mL	–34.95 (–72.47, 53.73)	–37.50 (–72.75, 41.91)
Q3: 6.2–18.1 ng/mL	–15.63 (–63.58, 93.48)	–17.30 (–62.09, 80.40)
Q4: > 8.1 ng/mL	–10.42 (–52.29, 158.57)	–16.18 (–51.32, 177.32)
*p*-Trend^*c*^	0.02	0.01
Male adolescents
Q1: ≤ 2.6 ng/mL	Reference	Reference
Q2: 2.7–5.7 ng/mL	–32.97 (–63.94, 23.37)	–24.42 (–55.51, 28.40)
Q3: 5.8–19.0 ng/mL	–39.95 (–61.71, –5.82)	–36.87 (–60.94, 2.02)
Q4: > 19.0 ng/mL	–10.42 (–41.73, 39.10)	–9.52 (–39.35, 33.64)
*p-Trend*^*c*^	0.04	0.08
Female children
Q1: ≤ 2.3 ng/mL	Reference	Reference
Q2: 2.4–3.9 ng/mL	–11.31 (–28.82, 11.63)	22.14 (–34.30, 2.02)
Q3: 4.0–15.3 ng/mL	–32.97 (–52.29, –4.88)	–35.60 (–58.10, –1.00)
Q4: > 15.3 ng/ mL	–9.52 (–36.87, 28.40)	–17.30 (–56.83, 60.00)
*p-Trend*^*c*^	0.11	0.06
Female adolescents
Q1: ≤ 2.5 ng/mL	Reference	Reference
Q2: 2.6–7.3 ng/mL	–2.96 (–24.42, 24.61)	–3.92 (–27.39, 25.86)
Q3: 7.4–31.6 ng/mL	–4.88 (–30.93, 32.31)	–12.19 (–37.50, 24.61)
Q4: > 31.6 ng/mL	–11.31 (–34.30, 20.92)	–10.42 (–34.30, 22.14)
*p*-Trend^*c*^	0.69	0.88
^***a***^Adjusted for age, race/ethnicity, serum cotinine, urinary creatinine, total cholesterol, income, obesity, season of collection, time of venipuncture, and physical activity (for adolescents only). ^***b***^Adjusted for model 1 covariates plus natural log–transformed BP-3, BPA, and ∑PAR. ^***c***^*p*-Value for exposure quartiles modeled as an ordinal variable using integer values.		

***Parabens*** (Table 5). There were no statistically significant associations between TT and ΣPAR in any subgroup before or after adjustment for BP-3, BPA, and TCS, and no evidence of consistent trends with increasing quartiles of exposure.

**Table 5:** Percent differences (95% CI) in serum total testosterone by quartiles of ∑PAR exposure, National Health and Nutrition Examination Survey, 2011–2012.

∑PAR exposure	Model 1^*a*^	Model 2^*b*^
Male children
Q1: ≤ 4.4 ng/mL	Reference	Reference
Q2: 4.5–10.1 ng/mL	44.77 (–21.34, 169.12)	43.33 (–17.30, 148.43)
Q3: 10.2–40.0 ng/mL	–15.63 (–46.74, 32.31)	–14.79 (–42.88, 28.40)
Q4: > 40.0 ng/mL	10.52 (–37.50, 95.42)	16.18 (–36.24, 113.83)
*p*-Trend^*c*^	0.22	0.29
Male adolescents
Q1: ≤ 4.9 ng/mL	Reference	Reference
Q2: 5.0–10.2 ng/mL	–22.12 (–55.96, 39.10)	–13.06 (–51.36, 53.73)
Q3: 10.3–61.5 ng/mL	–16.47 (–45.66, 27.12)	–3.92 (–32.29, 36.34)
Q4: > 61.5 ng/mL	–23.66 (–51.32, 18.53)	–14.79 (–48.31, 41.91)
*p*-Trend^*c*^	0.49	0.82
Female children
Q1: ≤ 11.3 ng/mL	Reference	Reference
Q2: 11.4–22.7 ng/mL	13.88 (–12.19, 47.70)	19.72 (–12.19, 63.23)
Q3: 22.8–76.8 ng/mL	12.55 (–21.34, 50.68)	18.53 (–13.06, 63.23)
Q4: > 76.8 ng/ mL	31.00 (–4.88, 82.21)	53.73 (–18.13, 191.54)
*p*-Trend^*c*^	0.39	0.11
Female adolescents
Q1: ≤ 15.5 ng/mL	Reference	Reference
Q2: 15.6–66.6 ng/mL	–8.61 (–34.95, 29.69)	–5.82 (–32.29, 31.00)
Q3: 66.7–297.9 ng/mL	–21.34 (–43.45, 8.33)	–24.42 (–44.57, 3.05)
Q4: > 297.9 ng/mL	3.05 (–29.53, 52.20)	4.08 (–25.92, 47.70)
*p*-Trend^*c*^	0.11	0.02
^***a***^Adjusted for age, race/ethnicity, serum cotinine, urinary creatinine, total cholesterol, income, obesity, season of collection, time of venipuncture, and physical activity (for adolescents only). ^***b***^Adjusted for model 1 covariates plus natural log–transformed BP-3, BPA, and TCS. ^***c***^*p*-Value for exposure quartiles modeled as an ordinal variable using integer values.		

## Discussion

To our knowledge, this is the first study to investigate the associations of BPA, BP-3, TCS, and ΣPAR with serum TT in children and adolescents. In this cross-sectional analysis of data from NHANES 2011–2012, both urinary BPA and urinary BP-3 levels were inversely associated with serum TT in male adolescents; conversely, higher levels of BPA were associated with higher serum TT in female adolescents. No significant associations were observed in children (male or female). Overall, there was little evidence of associations between TCS and TT, and no significant associations with ΣPAR in any of the four population subgroups.

Associations between BPA levels and TT differed between male and female adolescents, with an inverse association in males and a positive association in females. As reviewed by Gore et al. (2015), several animal studies have focused solely on the associations of BPA with serum testosterone in male animals; these studies have consistently reported decreased serum testosterone levels in these animals. However, other studies have looked at both male and female animals, and several of these studies have reported sex differences in testosterone levels following BPA exposure. Chen F et al. (2014) reported significantly lower serum testosterone in male rats exposed to BPA than in controls, whereas BPA-exposed female rats had nonsignificantly higher serum testosterone. Fernández et al. (2010) reported increased serum testosterone and serum estradiol levels in female rats exposed postnatally to BPA. Furthermore, Xi et al. (2011) reported that the expression of several genes related to the hypothalamic–pituitary–gonadal (HPG) axis was increased in both male and female mice pups following BPA exposure; several hormonal changes including decreased testosterone synthesis in male pups and enhanced aromatase expression levels and estrogen synthesis in female pups were contemporarily observed, suggesting that the changes in gene expression may have affected downstream hormonal levels.

Levels of serum TT in men have a wide range of variation due to diurnal, weekly, and seasonal variations, episodic secretion, and glucose ingestion (Brambilla et al. 2009; Cunningham and Toma 2011). Diurnal variation results in peak serum TT levels in the early morning followed by a progressive decline to the lowest levels in the evening, which may be as little as 15% lower than peak morning levels, and as much as 50% lower in younger males (Paduch et al. 2014). Testosterone circulates in the body either bound to sex hormone–binding globulin (SHBG), albumin, or corticosteroid binding globulin (CBG), or in an unbound form (free). SHBG-bound T represents approximately 44% of TT and is tightly bound and unavailable to cells. Albumin-bound T and CBG-bound T represent approximately 50% and 4% of TT, and for both, T is weakly bound and dissociates rapidly. Free testosterone (FT) represents about 2–3% of TT (De Ronde et al. 2006). The term “bioavailable” T refers to the sum of the CBG-bound, albumin-bound, and free components, thus representing the T fraction available to cells. In the present study, we evaluated TT, which includes both bioavailable and bound forms.

Few epidemiological studies have examined relationships between BPA and sex hormones, and study populations have been limited to adults. Our finding of a positive association of BPA and TT in female adolescents is in agreement with the findings reported by Takeuchi and Tsutsumi (2002) and Takeuchi et al. (2004). Takeuchi and Tsutsumi (2002) investigated the relationship between BPA exposure and hormone levels in men (*n* = 11) and women (*n* = 30), showing positive correlations between serum BPA concentrations with TT and FT levels in both sexes. A significant, positive relationship was reported between circulating androgen concentrations and BPA exposure in a small study of 26 normal women and 47 women with ovarian dysfunction (Takeuchi et al. 2004). The inverse association of BPA and serum TT that we found in male adolescents may be consistent with epidemiological studies conducted in male adults (Meeker et al. 2010; Mendiola et al. 2010; Zhou et al. 2013). Recently, Zhou et al. (2013), in a cross-sectional study of 290 men with or without BPA exposure in the workplace, found that serum BPA was significantly associated with decreased FT levels, decreased free androgen index (FAI), and increased SHBG levels. Mendiola et al. (2010) examined urinary BPA and serum hormones in fertile men (*n* = 360) and found a significant inverse association between urinary BPA concentration and FAI concentration levels as well as a significant positive association between BPA and SHBG levels. In a subset of 167 men attending an infertility clinic, Meeker et al. (2010) found that urinary BPA concentrations were inversely associated with serum inhibin B, FAI, and estradiol/testosterone index (a marker of aromatase activity). Additionally, they found a positive association between urinary BPA with both follicle-stimulating hormone (FSH) and FSH/inhibin B ratio (a marker of Sertoli cell function). Conversely, several studies reported a positive association of BPA and testosterone in male adults (Galloway et al. 2010; Hanaoka et al. 2002; Takeuchi and Tsutsumi 2002). Hanaoka et al. (2002) reported that urinary concentrations of BPA were significantly higher in 42 exposed workers than in matched controls (*n* = 42) and that urinary BPA level was inversely correlated with serum FSH concentrations (Hanaoka et al. 2002). Galloway et al. (2010) found that higher daily BPA excretion was associated with a higher TT concentration in 307 men from the InCHIANTI study among adult males in Italy.

To our knowledge, the only epidemiological study investigating the association of urinary BP-3 with sex hormones was conducted by Janjua and colleagues (2004). The authors reported that the mean serum testosterone concentration in 15 young men was significantly lower 4 hr after they were treated with cream containing BP-3 compared with levels 4 hr after a control cream was applied. However, there was no significant difference 24 hr after application of the BP-3 or control cream. Testosterone levels were also lower in 17 postmenopausal women after application of the BP-3 versus control cream, but because testosterone levels were lower before and after the BP-3 cream was applied, the authors attributed the finding to chance.

The present study has several important limitations. First, the cross-sectional nature of this study limits the inferences that can be made based on the results. Moreover, 12- to 19-year-old boys and girls classified as adolescents may have included a mix of children who were pre- and postpubescent. Recently, Lopez-Espinosa et al. (2011), in their study of the association between sex hormones and perfluorocarbon compounds, considered TT > 50 ng/dL to be a cutoff for reaching puberty in male children/adolescents. Based on this cutoff, we found that at age 10 and at age 11 years, 7.59% and 29.17% of male participants had reached puberty, respectively; no male children < 10 years of age had reached puberty based on this cutoff point. On the other hand, for those 12 years and 13 years of age, 67.16% and 83.56% had reached puberty, respectively; all male adolescents > 13 years of age had serum TT levels higher than 50 ng/dL (data not shown). Therefore, the age cutoff used in this study to classify boys as children or adolescents seems appropriate.

Recent findings from experimental studies suggest that BPA may affect aromatase activity, which might subsequently lead to changes in concentrations of FT, FAI, or estradiol (E_2_), and in the E_2_/T ratio. For example, Castro et al. (2013) reported increased aromatase activity in adult male Wistar rats exposed to BPA, concurrent with decreased testosterone and increased E_2_ and E_2_/T ratio levels. E_2_ and aromatase activity increased in the urogenital sinus of fetal mice after maternal exposure to low-dose BPA (Arase et al. 2011). Furthermore, BPA-induced aromatase activation was reported by Kim et al. (2010) in rat testicular Leydig cells and by Nativelle-Serpentini et al. (2003) in human placenta JEG-3 cells following pre-incubation times ranging from 10 min to 6 hr, whereas Chen S et al. (2014) showed that BPA did not induce aromatase activation in human MCF-7 breast cancer cell lines, instead functioning as an estrogen receptor agonist. Conversely, other *in vitro* studies have reported aromatase inhibition by BPA (Benachour et al. 2007; Bonefeld-Jørgensen et al. 2007). These hormones were not measured for the 2011–2012 NHANES survey, so we were unable to investigate whether there was any association with BPA in the population of interest.

The use of a single spot measurement of serum TT is also a limitation given cyclical variation in hormone levels. We adjusted for the time of day (session) when the sample was collected (morning, afternoon, or evening) and for the season of collection (1 November–30 April or 1 May–31 October), but residual confounding is possible if the timing or season of sample collection was associated with exposure levels. Another important limitation is the use of single spot urinary phenols and parabens measurements. Because these chemicals are short lived in the body, a biomarker of longer-term exposure would have been better. It has been shown that a single sample of BPA may be predictive of long-term exposure; Mahalingaiah et al. (2008) reported that study participants were correctly classified as being in the highest tertile of BPA exposure about two-thirds of the time, based on data from 31 men and women attending an infertility clinic. However, overall, the use of the single spot measurements of exposure is a limitation of this study. There may also have been other confounding factors that we did not control for in our analyses, including exposure to other environmental chemicals that are potentially anti-androgenic, such as phthalates (Meeker and Ferguson 2014).

## Conclusion

In conclusion, urinary levels of BP-3 and BPA were associated with lower levels of serum testosterone in male adolescents (age 12–19 years), while BPA was positively associated with serum TT in female adolescents in NHANES 2011–2012. These findings are of interest due to the pervasiveness of these chemicals in our environment and the potential impact that altered reproductive hormones, such as testosterone, can have on the development and overall health of children and adolescents. Because of the limitations of this study, our results should be interpreted with caution. However, our results support the need for future studies to confirm our findings and investigate potential mechanisms for differences between males and females, including longitudinal observational studies of associations between long-term exposures to EDCs and endocrine and reproductive hormone levels, and experimental animal and *in vitro* studies of the effects of low-dose environmentally relevant exposures.
